# Exosomal miR-186 derived from BMSCs promote osteogenesis through hippo signaling pathway in postmenopausal osteoporosis

**DOI:** 10.1186/s13018-020-02160-0

**Published:** 2021-01-07

**Authors:** Lu Li, Xin Zhou, Jun-tao Zhang, Ai-feng Liu, Chao Zhang, Jin-chang Han, Xiao-qing Zhang, Si Wu, Xiao-yu Zhang, Fu-quan Lv

**Affiliations:** 1 Department of Acupuncture, Tianjin Nankai Hospital, Tianjin, 300100 People’s Republic of China; 2 Department of Orthopedics, First Teaching Hospital of Tianjin University of Traditional Chinese Medicine, Tianjin, 300381 People’s Republic of China

**Keywords:** Postmenopausal osteoporosis, Exosomes, miRNA, Osteogenesis

## Abstract

**Background:**

Postmenopausal osteoporosis (PMO) that results from estrogen withdrawal is the most common primary osteoporosis among older women. However, little is known about the mechanism of PMO, and effective treatment of PMO is limited.

**Methods:**

We used real-time polymerase chain reaction (qPCR), Western blotting, and RNA pull down to investigate the relationship between miR-186 and MOB Kinase Activator 1A (Mob1). Also, we investigated the effect of exosome in osteogenesis using alkaline phosphatase (ALP) staining. And hematoxylin eosin (HE) staining was used to verify the osteogenesis in PMO model.

**Results:**

Exosomal miR-186 plays an important role in bone formation. The results of miRNA-seq and q-PCR showed that miR-186 was upregulated in a PMO + Exo treatment group. Results of RNA-pull down and luciferase reporter assays verified interactions between miR-186 and Mob1. We also verified the Hippo signaling pathway plays an important role in osteogenesis.

**Conclusions:**

We concluded that exosomes derived from human bone marrow mesenchymal stem cells (hBMSCs) can transfer miR-186 to promote osteogenesis in ovariectomy (OVX) rats through the Hippo signaling pathway.

## Introduction

Postmenopausal osteoporosis (PMO) resulting from estrogen withdrawal is the most common primary osteoporosis disease among older women. PMO is characterized by low bone mass and microarchitectural changes of cancellous bone with low bone mineral density (BMD) (i.e., T scores less than −2.5) and subsequently increased susceptibility for osteoporotic fractures. Osteoporotic fractures are the most common complication resulting from PMO. Approximately 50-year-old white woman has a 15 to 20% lifetime risk of hip fracture [1]. Approximately 1.5 million osteoporotic fractures per year caused by PMO occurred in the USA [2]. More than 40% of Caucasian postmenopausal women are affected by osteoporosis; moreover, the number of postmenopausal women is expected to increase in the near future with increasingly aging populations [1, 3]. However, little is known about the mechanism of PMO, and effective treatment is limited. A randomized clinical trial conducted by Hinton et al. showed that muscle mass and BMD can be improved with resistance and weight-bearing exercise [4]. Calcium and vitamin D treatments for preventing osteoporotic fractures have also been widely used in clinics. However, the effects of calcium and vitamin D treatments remain controversial. In a large randomized trial, Women’s Health Initiative investigators showed no significant effects on fractures in more than 36,000 postmenopausal women on calcium and vitamin D [5]. Some drugs regulating bone remodeling, including bisphosphonates, denosumab, and teriparatide, showed positive effects on increasing bone formation; however, the costs of these drugs were too expensive and increased financial burdens. Therefore, there is an urgent need to determine the pathological mechanism of PMO to develop more effective therapeutic strategies.

Exosomes are 30- to 150-nm diameter extracellular vesicles [6]. Exosomes contain endosome-derived components, including miRNA, non-coding RNA (ncRNA), and proteins [7, 8]. Recent studies have concluded that exosomes derived from stem cells can promote tissue regeneration in skin, cartilage, bones, and muscles [8–11]. Xiao et al. reported that cardiac progenitor cell-derived exosomal miR-21 can decrease oxidative stress to protect myocardium by targeting Programmed Cell Death 4 (PDCD4) [10]. Kuang et al. showed that exosomes derived from Wharton’s jelly of human umbilical cord mesenchymal stem cells can transfer miR-21-5p and significantly reduce osteocyte apoptosis for the treatment of glucocorticoid-induced osteonecrosis of femoral heads in rats by activating the AKT serine/threonine kinase (AKT) signaling pathway [12]. Liu et al. reported that stem cell-derived exosomes can promote cartilage regeneration [13]. Although the effects of exosomes have been explored in tissue regeneration, few studies have investigated the molecular mechanisms of exosomes in bone metabolism, especially during PMO. As previously reported, the abnormal expansion of marrow adipose tissue and osteoblast proliferation and differentiation are key factors that promote the progression of PMO [14]. In this study, we hypothesized that exosomes derived from bone marrow mesenchymal stem cells (BMSCs) may promote osteoblast proliferation and differentiation to inhibit PMO progression. Further, we hypothesized that exosomal miR-186 plays an important role in bone formation.

## Material and methods

### Animal experiments

All animal procedures were performed according to the National Institutes of Health Guide for the Care and Use of Laboratory Animals. The Animal Care and Use Committee of Nankai Hospital approved this experimentation. We used forty 294 ± 11 g female 8-week-old Sprague Dawley (SD) rats. As previously reported, the PMO model was induced by a rat model of osteoporosis using ovariectomy (OVX) for 2 months [15]. Rats with OVX were randomly and equally divided into four groups: (1) OVX + PBS group; (2) OVX + exosomes group; (3) OVX + exosomes + miR-186 inhibitor; and (4) OVX + miR-186 mimics. One hundred microliter (about 10^13^/mL) exosomes were used for the treatment of OVX rats with tail vein injection once a week. And 50 μM miR-186 mimics or inhibitor were used in OVX rats with tail vein injection once a week. When the rats were treated with different interventions for 28 days, all the rats between groups were sacrificed for experiment. Cervical dislocation was used for rat’s euthanasia. And 10% 350 mg/kg chloral hydrate (intraperitoneal injection) was used for anesthesia. Bones of the lower extremities were collected for subsequent experiments.

### Cell culture and treatments

Human bone marrow mesenchymal stem cells (hBMSCs) were donated by Professor Xin-long Ma from Tianjin Hospital and cultured for exosome extraction. BMSCs from the bone marrows of OVX or normal rats were also extracted for cellular experiments. The BMSCs were incubated in α-MEM (α-MEM, Gibco, Paisley, UK) medium and supplemented with 10% fetal bovine serum (FBS, HyClone, Logan, UT) and 100 μg/ml penicillin/streptomycin at 37 °C.

The BMSCs from OVX rats were co-cultured with hBMSC exosomes. CCK-8, RT-PCR, Western blotting, and RNA-seq assays were performed using the cultured cells.

### Exosome isolation, purification, and identification

Exo-free FBS was prepared by ultracentrifugation at 130,000×*g* for 18 h; FBS supernatant was collected [16]. When hBMSC cultures reached 60-70% confluence under normal culture conditions, exo-free FBS was changed for exosome collection. Next, hBMSC supernatant was collected for another 48 h. Then, exosomes derived from hBMSCs were extracted using exosome extraction kits (exoEasy Maxi Kit, QIAGEN, Germany). Exosome morphology derived from hBMSCs was detected by transmission electron microscopy (Hitachi H-7650, Japan). We used dynamic light scattering (DLS, Nicomp 380, USA) to determine exosome size distribution. Specific exosome biomarkers, including Alixs, CD63, and CD81, were analyzed by Western blot.

### Exosome labeling with PKH-26

We used PKH-26 (Sigma-Aldrich) to label extracted exosomes. Exosomes extracted from hBMSCs were co-cultured with PKH-26 mixture for 15 min under dark. Next, 10% BSA was used to stop the staining reaction for 1 min. Labeled exosomes were extracted by ultracentrifuge at 100,000×*g* for 1 h at 4 °C.

### Cell viability assay

Cell Counting Kit-8 (CCK-8, Beyotime Biotechnology, China) assays were used to calculate the proliferation rates of BMSCs from OVX or normal rats. Extracted BMSCs were seeded in 96-well plates at 2000 cells per well for 3 days. Then, 10 μL CCK-8 was added in each well and incubated for 4 h under dark. An enzyme-linked immunosorbent assay reader was used to detect OD values at 450 nm.

### RNA extraction, reverse transcription, and q-PCR

Trizol reagent (Invitrogen, Carlsbad, CA, USA) was used for total RNA extraction. A miRNA purification kit was used for miRNA separation. Reverse transcription of cDNA was performed according to manufacturer instructions. Real-time PCR was performed using a PrimeScript RT reagent kit and detected by Applied Biosystems model 7900HT Fast Real-Time PCR System (Thermo Fisher, USA). The 2^−△△Ct^ method was used for gene expression calculation [17].

### High-throughput miRNA sequencing

High-throughput sequencing was performed on a BGUSEQ-500 by BGI (Beijing Genomics institution, China), and miRNA expression between the PMO and PMO + exosomes groups were detected. First, the hind limbs of rats in the PMO and PMO + exosomes groups were harvested and frozen using liquid nitrogen. Then, a BioAnalyzer 2100 system was used to quantify the quality of extracted miRNA. Clean tags of miRNA were mapped to a reference genome library. Differentially expressed miRNA was analyzed and defined by the bioinformatics service of BGI with FDR < 0.05 and *p* < 0.05.

### RNA pull-down assay

We performed an RNA pull-down (Thermo Fisher, USA) assay with biotinylated miR-186 to detect the relationship between the miR-186 and Mob. BMSCs were transfected with biotinylated miR-186 mimics or biotin-negative control (50 nM) and harvested at 48 h after transfection. The biotin-coupled RNA complex was pulled down by incubating the cell lysates with streptavidin-coated magnetic beads. The abundance of ceRNA in the bound fractions was evaluated by qRT-PCR analysis.

### Osteogenic differentiation assay

BMSCs in different groups were cultured in six-well plates containing osteogenic differentiation medium (Cyagen, Suzhou, China). Media were changed every 3 days for 3 weeks. After rinsing, cells were fixed with 4% paraformaldehyde. Mineralized nodules were observed by staining with a 1% alizarin red solution (Cyagen, Suzhou, China) following manufacturer instructions [12]. ALP staining was used to investigate the osteogenesis of BMSCs after induction [18]. Staining images were recorded under an inverted microscope, and ImageJ was used to analyze the results.

### Hematoxylin and eosin staining

Paraformaldehyde (Beyotime Biotechnology, China) was used to fix tibiae from different rats for a week. The fixed samples were washed three times to remove excess paraformaldehyde; then, the tibiae were embedded in paraffin and cut into 5-μm sections. The sections were deparaffinized in xylene and rehydrated through a graded series of ethanol. Hematoxylin and eosin (HE, Solarbio, China) staining was performed according to manufacturer instructions. Microscopy was used to observe the morphology of cancellous bone in the tibia. We used the left hind leg of rats for HE staining, and all the groups tested on the same side.

### Transfection of miRNA mimics and inhibitor

According to manufacturer instructions, miR-186 mimics and inhibitors and negative control (50 nM) were purchase from Sangon Biotech and transfected into BMSCs using Lipofectamine™ 2000 Transfection Reagent (Thermo Fisher, USA). Target gene expressions were evaluated using RT-PCR; luciferase intensity was measured using a Dual-Luciferase Reporter Assay System 48 h post-transfection. qPCR was used to evaluate the transfection efficiency of miR-186 mimics and inhibitor. The negative control of miR-186 mimics was sense UUCUCCGAACGUGUCACGUTT, antisense ACGUGACACGUU CGG AGA ATT. The negative control of miR-186 inhibitor was CAGUACUUUUGUGUAGUACAA.

### Western blotting assay

Protein from cells or bone tissues were extracted using RIPA buffer. Then, 10% SDS-PAGE was used to separate protein lysates. Proteins were electrophoretically transferred to a 0.22-μm polyvinylidene difluoride membranes (PVDF, Millipore, USA). The membranes were blocked with 5% milk-TBST for 1 h at room temperature and incubated overnight at 4 °C with primary antibodies against Bone Morphogenetic Protein 2 (BMP2, Abcam, ab214821), YAP (CST, 14074), and β-Actin (CST, 3700) at a 1/1000 dilution. The membranes were then incubated for 1 h at room temperature with HRP-linked antibody at a 1/5000 dilution, and TBST was used to wash the membrane. The band was detected by chemiluminescence and analyzed using the ImageJ software.

### Dual-luciferase reporter assay

PCR product containing the 5′-flanking sequence of the MOB1 promoter was first inserted into a pGL3-basic vector. Then, 200 ng pGL3-MOB1 Promoter, along with 40 ng pRL-TK Vector (E2241, Promega) were respectively transfected into BMSCs using Lipofectamine 2000 for 48 h. And then, the Dual-Luciferase Reporter Assay System was used to detect luciferase intensity. Firefly luciferase activity was normalized to Renilla luciferase activity for each transfected well.

### MicroCT analysis

The tibia in rats with different interventions were obtained and fixed with 4% paraformaldehyde. And then, we used the microCT manufactured by Siemens (Berlin, Germany) to reconstruct the bone structure and calculate the bone parameters with Inveon analysis workstation. The bone parameters including bone volume/total volume (BV/TV), trabecular thickness (Tb.Th), trabecular number (Tb.N), and trabecular separation (Tb.Sp) were used for bone quality calculation.

### Statistical analysis

SPSS 19.0 statistical software package was used to perform statistical analyses. Analysis of variance was used to detect significant differences between groups. Post hoc multiple comparison test (Fisher’s least significant difference [LSD] test and Tukey’s test) was performed for the statistical analysis between every two groups. We used the two independent samples *t* test to evaluate results between two groups. Results are reported as means ± SD. Two-tailed *p* values 0.05 or less were considered significant.

## Results

### Characterization of exosomes derived from hBMSCs

**Fig. 1:**
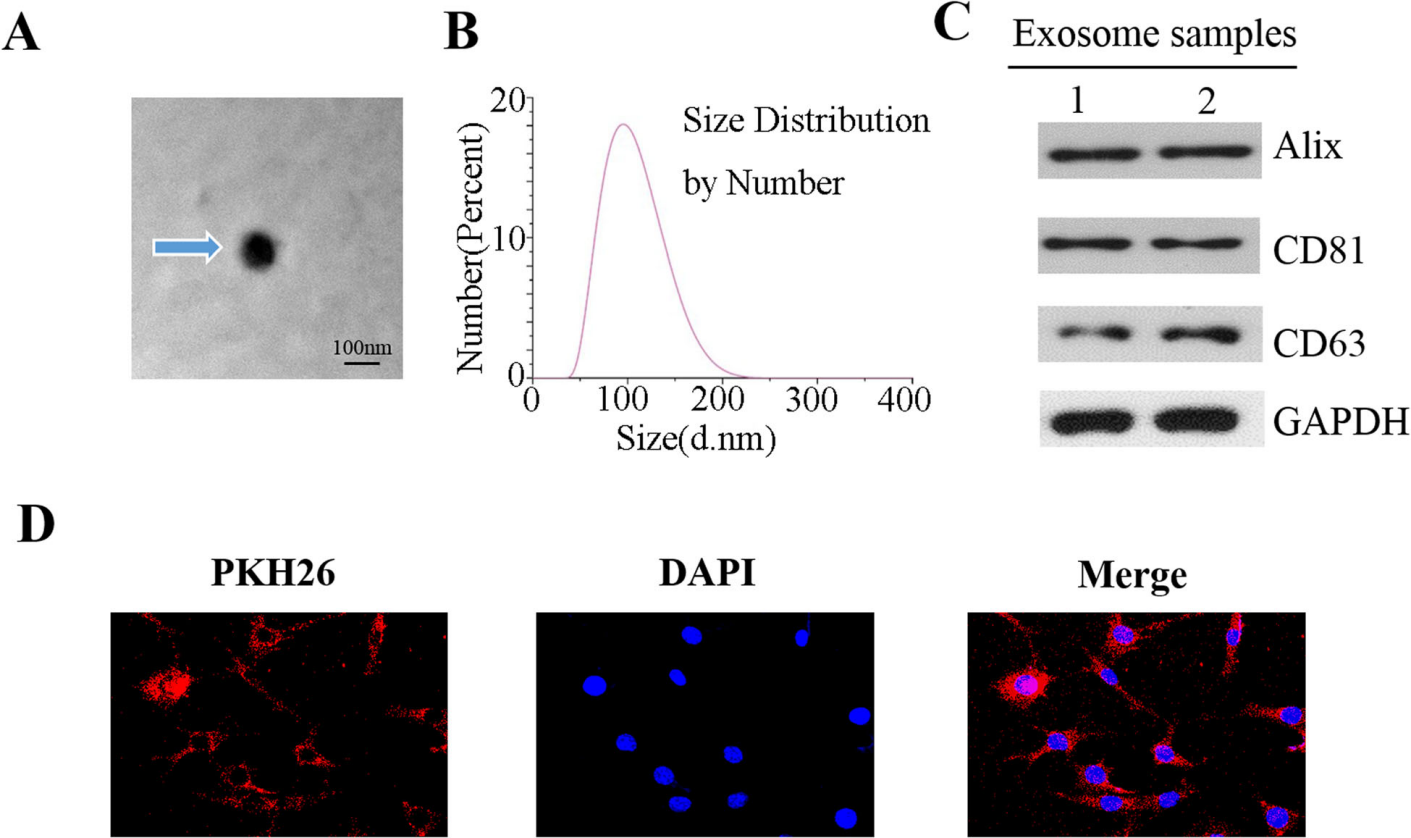
**a** The morphology of exosomes derived from hBMSCs were detected using transmission electron microscopy. Phosphotungstic acid staining was used before exosome identification. **b** The size distribution of exosomes was identified using DLS. **c** The surface biomarkers of exosomes, including Alixs, CD81, and CD63 were detected using Western blot. **d** The exosomes were labeled with PKH26, and the labeled exosomes were observed using confocal microscopy

First, we used TEM, DLS analysis, and Western blot to identify purified exosomes derived from hBMSCs. The TEM results showed that exosomes derived from hBMSCs comprised a double layer phospholipid membrane structure with a 100-nm-diameter round shape (Fig. 1a). The DLS analysis results verified the size distribution, which predominantly ranged from 100 to 150 nm (Fig. 1b). Western blot detection of exosome-related markers showed that CD63, CD81, and Alix were significantly expressed in exosomes derived from hBMSCs (Fig. 1c). PKH26 staining was used to label exosome membranes; labeled exosomes were observed in BMSCs using confocal microscopy (Fig. 1d).

### Exosomes derived from hBMSCs promoted osteogenesis and cell proliferation

**Fig. 2:**
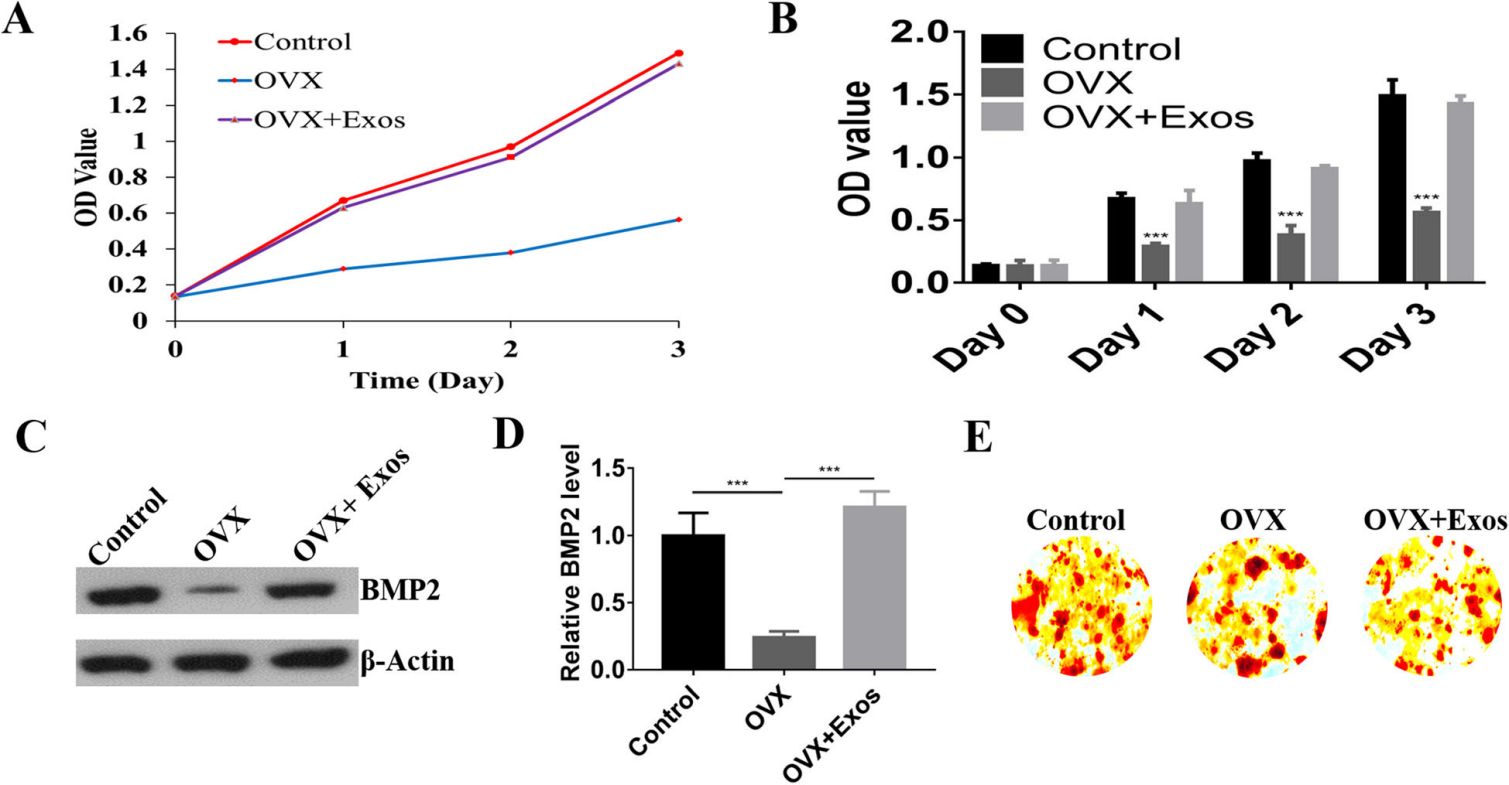
**a** CCK-8 assay was conducted to detect the proliferation rates of BMSCs extracted from OVX rats. **b** The CCK-8 results were calculated using the SPSS software. **c** Bone metabolism markers, including BMP2 and RANKL, were detected using Western blot. **d** The expressions of BMP2 were calculated using ImageJ. **e** The alizarin red staining was performed to detect the osteogenesis effect of exosomes

Next, we investigated the cell proliferation and osteogenesis of exosomes derived from hBMSCs. The proliferation rate of BMSCs from the different groups, i.e., normal cells, OVX, and OVX + exosomes, was evaluated by CCK-8. The results showed that the proliferation rates of BMSCs from OVX models were significantly inhibited and that exosomes can rescue the OVX effect compared with controls (Fig. 2a and b). We explored the effects of exosomes on osteogenesis using Western blot to detect the expression of bone metabolism markers, including BMP2. BMP2 expression was significantly inhibited in the OVX group, and BMP2 was activated with exosome treatment (Fig. 2c and d). Also, the alizarin red staining was performed. The results showed that exosomes can promote the osteogenesis (Fig. 2e).

### Exosomal miR-186 regulates osteogenesis by targeting Mob1

**Fig. 3:**
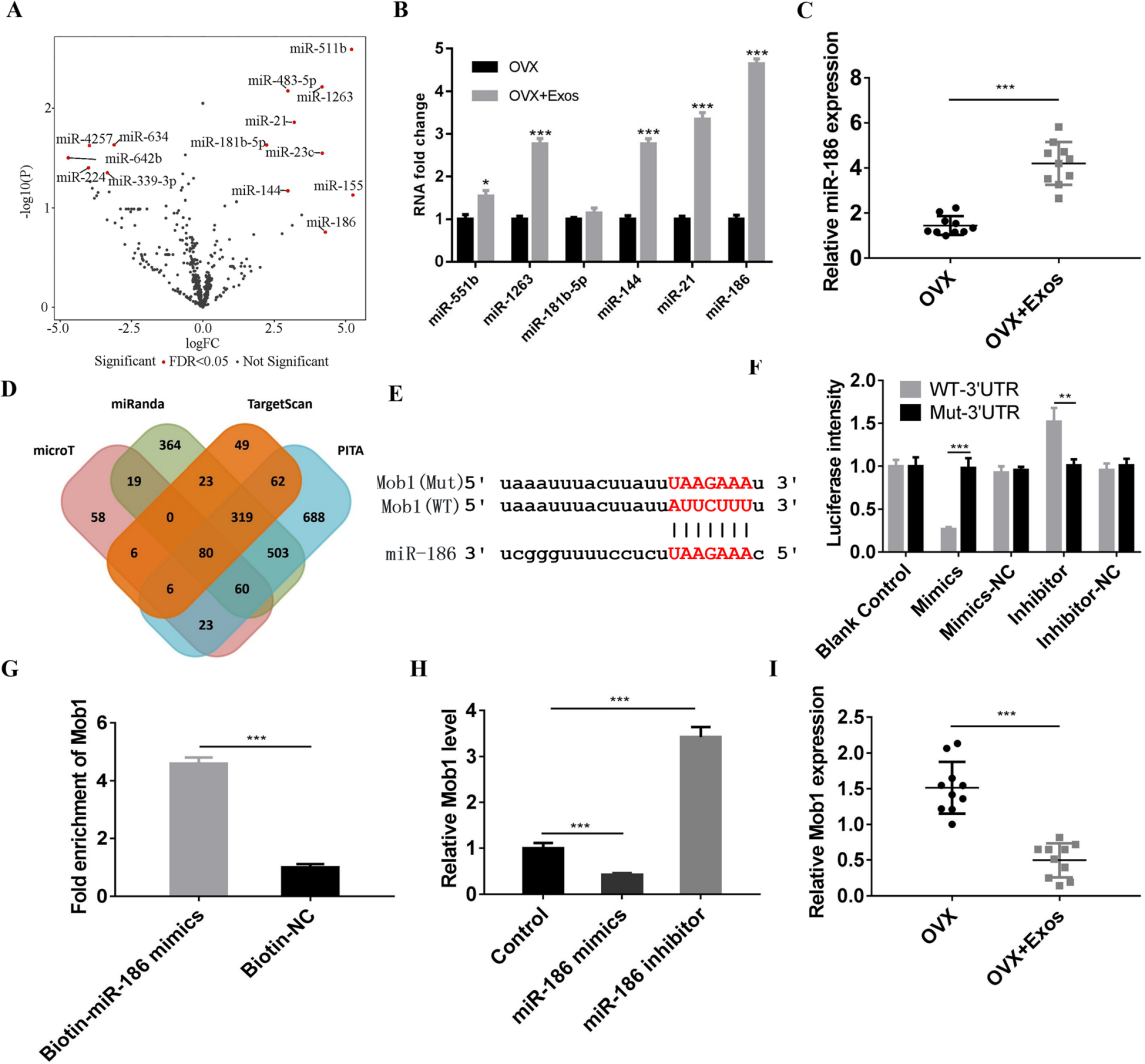
**a** The differentially expressed miRNAs were detected between BMSCs extracted in OVX rats (PMO) and BMSCs extracted in OVX rats + exosomes groups (PMO + Exos) using miRNA-seq. **b** q-PCR was performed to investigate the expression of miRNA between PMO and PMO + exosome group. **c** RNA in tibia of rats were extracted and the expression of miR-186 in tibia of rats were verified using q-PCR. **d** microT, miRanda, Target Scan, and PITA were used to predict the downstream targets of miR-186. **e** The binding sites between Mob1 and miR-186 were shown, and mutant or wild-type nucleotides were labeled in red. **f** The relationship between Mob1 and miR-186 was verified using the luciferase reporter assay. **g** RNA-pull down assays were conducted to further verify the interaction between Mob1 and miR-186. **h** q-PCR was performed to detect the expression of Mob1. **i** The expression of Mob1 was detected between the PMO and PMO + Exos groups using q-PCR

miRNA-seq was performed to investigate the molecular mechanisms of exosomes derived from hBMSCs on osteogenesis; related differentially expressed miRNAs in exosomes were also detected. The results showed that nine miRNAs were upregulated and five miRNAs were downregulated in the exosomes+OVX group (Fig. 3a). We verified the expressions of miR-551b, miR-1263, miR-181b, miR-144, miR-21, and miR-186 using q-PCR. These miRNAs were all upregulated in the exosomes+OVX group; miR-186 had higher expression in the exosomes+OVX group than in the OVX group (Fig. 3b). Additionally, we verified the results of q-PCR using bone samples in rats; miR-186 was also upregulated in the bone tissues of exosomes+OVX rats (Fig. 3c).

Next, we investigated the molecular mechanism of exosomal miR-186 for promoting osteogenesis of BMSCs from OVX rats. We first explored potential genes targeted by miR-186. Online electronic databases containing PITA, microT, Target Scan, and miRanda were used for downstream target searching (Fig. 3d). As previously reported, the Hippo signaling pathway is responsible for regulating cell differentiation, apoptosis, osteogenesis, and proliferation [19, 20]. Mob1 is an important gene in the Hippo signaling pathway in regulating the expression of downstream genes, including LATS and YAP/TAZ [21, 22]. Among these candidate target genes, we determined the binding site between Mob1 and miR-186 (Fig. 3e). To explore the relationship between Mob1 and miR-186, we designed the luciferase assay to verify the binding site between Mob1 and miR-186. The results showed that the miR-186 mimics decreased luciferase intensity, whereas miR-186 inhibitor increased luciferase intensity; however, the mutant Mob1 had no significant effect on luciferase intensity (Fig. 3f). In addition, RNA-pull downs were performed to further verify interactions between Mob1 and miR-186. The enrichment of Mob1 in the biotin-miR-186 group was significantly higher than the biotin negative control group (Fig. 3g). Moreover, we explored the expression of Mob1 when transfected with miR-186 mimics or inhibitor. miR-186 mimics inhibited the expression of Mob1; miR-186 inhibitor promoted the expression of Mob1 (Fig. 3h). Bone samples were used to verify the expression of Mob1; the results showed that the expression of Mob1 decreased in the OVX + exosomes group (Fig. 3i).

### Exosomal miR-186 regulated osteogenesis through hippo signaling pathway in OVX rat model

**Fig. 4:**
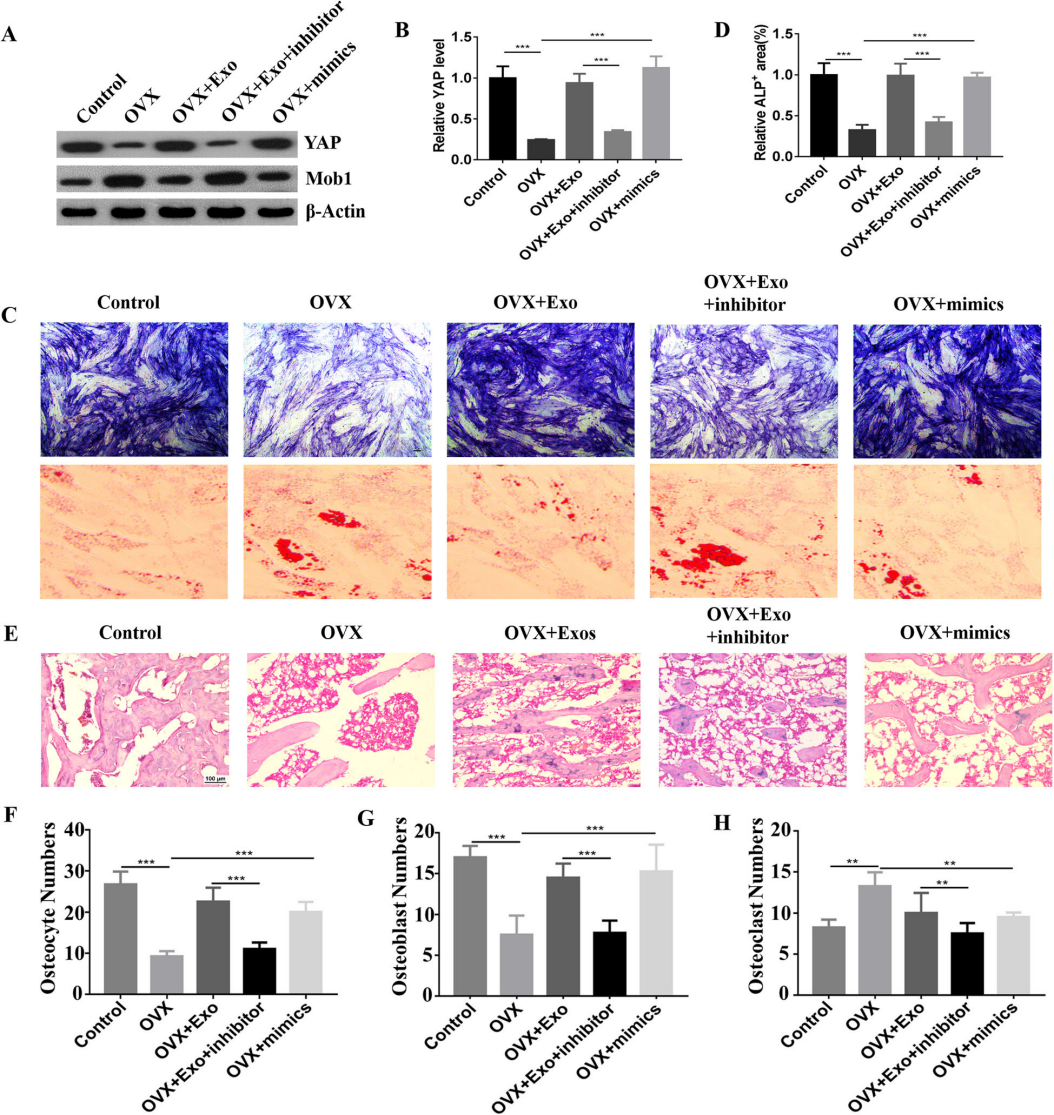
**a** The expression of YAP was detected in the control group, PMO group, PMO + Exo group, PMO + Exo + miR-186 inhibitor group, and PMO + miR-186 mimics group. **b** YAP expression was calculated using ImageJ. **c** ALP staining and oil red staining was performed to detect osteogenesis and adipogenesis between different groups. **d** Quantitative calculation of ALP-positive areas. **e** HE staining of tibia in rats receiving different treatments was performed to evaluate the osteogenesis of exosomal miR-186. **f** The number of osteocytes were calculated. **g** The number of osteoblasts was calculated. **h** The number of osteoclasts was calculated

**Fig. 5:**
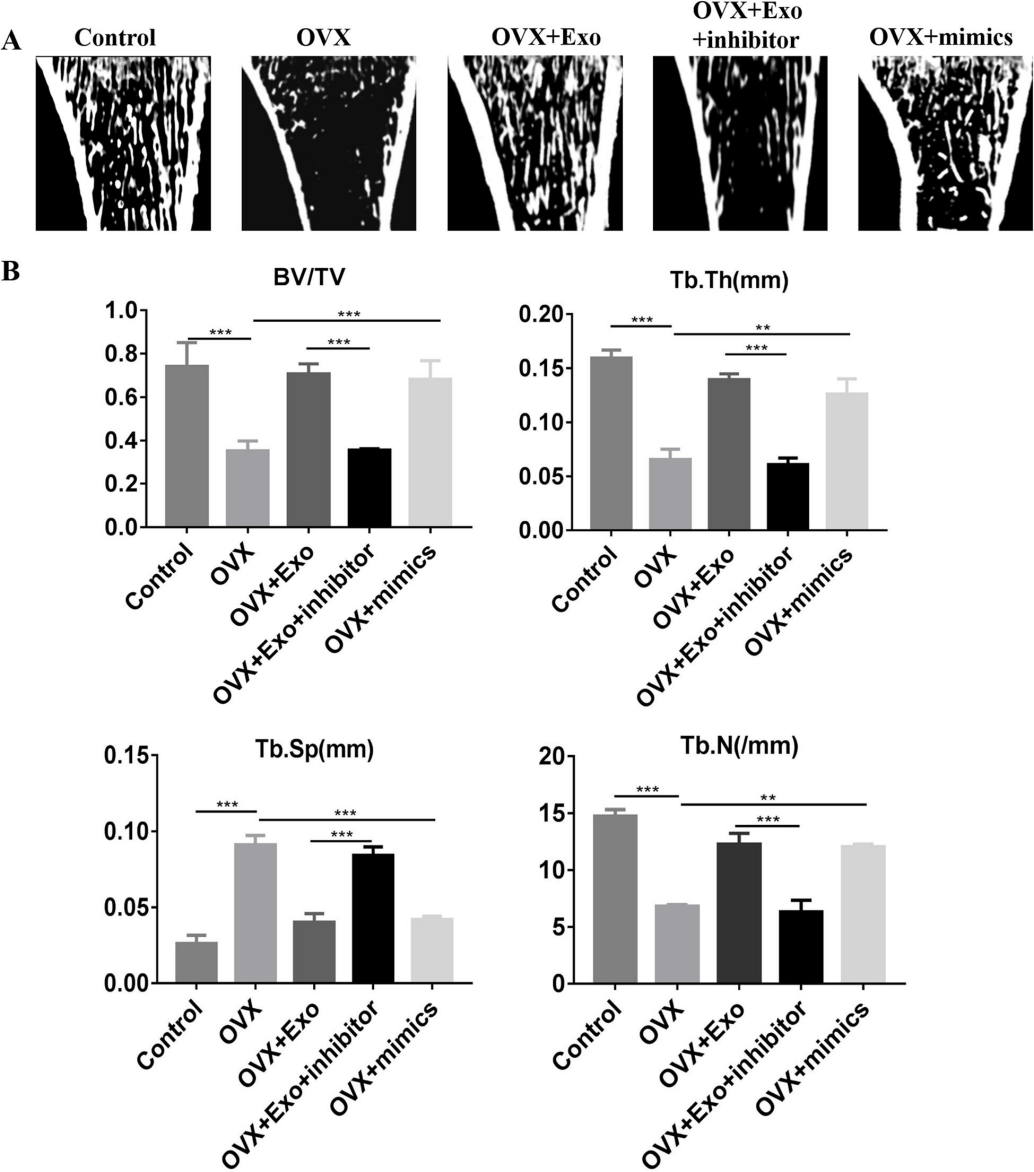
**a** The microCT was used to evaluate the bone mass between different groups. **b** The bone parameters were calculated between different groups

To investigate the functional roles of exosomes on PMO, an OVX rat model was prepared by ovariectomy. Western blot was performed to investigate the expression of YAP when BSMCs were treated with exosomes or miR-186 mimics or inhibitor. Exosomes and miR-186 mimics promoted the expression of YAP, whereas miR-186 inhibitor decreased the expression of Yes1 Associated Transcriptional Regulator (YAP) and Mob1 (Fig. 4a and b). Additionally, we performed ALP staining to investigate the osteogenesis of exosomes. Exosomes and miR-186 mimics increased ALP expression, whereas miR-186 inhibitor inhibited ALP expression (Fig. 4c and d). Also, the adipogenesis was evaluated using oil red staining. The results showed that the exosomes and exosomal miR-186 can inhibit the adipogenesis (Fig. 4c). The results of HE staining in tibiae showed no significant osteoporosis in the normal group; however, osteoporosis was well observed in the OVX group (Fig. 4e). Exosomes and miR-186 mimics were observed to reverse osteoporosis caused by OVX, whereas miR-186 inhibitor promoted osteoporosis (Fig. 4e). We also calculated the numbers of osteocytes (Fig. 4f), osteoblast (Fig. 4g), and osteoclast (Fig. 4h) between different groups, the results showed that exosome can promote the cell numbers of osteocytes and osteoblasts, but the cell numbers of osteoclasts were inhibited. In addition, we performed microCT analysis to detect the bone mass. The results showed that the bone volume decreased in the OVX group when compared with the control and OVX + Exos group, and the miR-186 mimics were observed to increase the bone volume, whereas miR-186 inhibitor decreased the bone volume (Fig. 5a and b).

## Discussion

PMO should be managed to prevent osteoporotic fractures [1, 2]. PMO incidence has steadily increased with the aging of the postmenopausal women population [23]. Approximately 50% of postmenopausal women are affected by PMO, and nearly 40% of osteoporotic patients suffer from osteoporotic fractures [14]. Therefore, there is an urgent need to further clarify the pathogenesis of PMO to determine new treatment strategies. BMSCs are important precursors of osteoblastic-lineage cells and are a main source of osteoblasts. Previous studies have verified that abnormal bone marrow adipose tissue was accompanied with bone loss in PMO and OVX animals, which indicated that the number and stem cell properties of BMSCs are important factors in regulating PMO [24, 25]. Previous studies have demonstrated exosomes derived from stem cells showed potential in promoting tissue regeneration [26–28]. Therefore, we used exosomes derived from hBMSCs for the treatment of PMO in vivo and in vitro. We found that exosomal miR-186 was the key miRNA to regulate osteogenesis in PMO.

Although PMO mechanisms have been explored in detail, PMO treatment protocols have primarily focused on anti-osteoporotic drugs, including diphosphonate, teriparatide, and denosumab [2, 3]. The mechanisms of these drugs include osteogenesis promotion and osteoclast inhibition. However, the economic burden limits the clinical applications of these drugs. Exosomes derived from stem cells have been identified as the main trophic factors that regulate cell proliferation and differentiation [8]. It has been shown that exosomes derived from stem cells promote osteogenesis [29]. In our study, we extracted exosomes from hBMSCs; these exosomes significantly increased the expression of bone metabolic markers, such as BMP2 and RANKL, which indicated that exosomes had the potential to promote osteogenesis.

As previously reported, exosomal miRNAs play important roles in regulating cellular functions [30]. Emily et al. [31] reported exosomal miR-21 was an important marker in traumatic brain injury through miRNA-seq. Yang et al. [32] performed RNA-seq and found miR-1263 prevented BMSC apoptosis in disuse osteoporosis. In a study conducted by Kuang et al., exosomes derived from human umbilical cord mesenchymal stem cells transferred miR-21 to regulate osteogenesis in GIONFH [12]. In our study, we extracted BMSCs from OVX or normal rats, and we performed miRNA-seq between BMSCs from OVX and BMSCs from OVX + exosomes groups; differentially expressed miRNAs were explored. We found that miR-186 was upregulated in the exosome-treated groups. We verified exosomal miR-186 can promote osteogenesis in vitro and in vivo. This is the first study to report that the molecular mechanism of exosomal miR-186 for the treatment of PMO.

We also investigated the downstream targets of miR-186. Four databases were referenced to predict the target mRNA. As previously reported, the Hippo signaling pathway plays an important role in cell proliferation and differentiation [22, 33, 34]. We searched related mRNA in the databases and found that Mob1 is a potential target of miR-186 and is a co-factor in regulating YAP [35]. Therefore, we chose Mob1 as the target and verified the relationship between miR-186 and Mob1 using q-PCR, RNA-pull down, and luciferase reporter assays. Western blot assays showed that exosomes promoted the expression of YAP, whereas miR-186 inhibitor decreased the expression of YAP. The OVX rat models were used to verify the effects of exosomes and exosomal miR-186. HE staining was performed to explore bone morphology; the results were consistent with those of the ALP staining.

## Conclusions

Therefore, we concluded that exosomes derived from hBMSCs transferred miR-186 to promote osteogenesis in OVX rats through the Hippo signaling pathway.

## Data Availability

All the data and materials are available for publication.

## Abbreviations

PMO: Postmenopausal osteoporosis
OVX: Ovariectomy
BMD: Bone mineral density
BMSCs: Bone marrow mesenchymal stem cells
CCK-8: Cell Counting Kit-8
HE: Hematoxylin and eosin
qPCR: Real-time polymerase chain reaction
ALP: Alkaline phosphatase
DLS: Dynamic light scattering
ncRNA: Non-coding RNA
